# CCN5 activation by free or encapsulated EGCG is required to render triple‐negative breast cancer cell viability and tumor progression

**DOI:** 10.1002/prp2.753

**Published:** 2021-03-21

**Authors:** Amlan Das, Inamul Haque, Priyanka Ray, Arnab Ghosh, Debasmita Dutta, Mohiuddin Quadir, Archana De, Sumedha Gunewardena, Indranil Chatterjee, Snigdha Banerjee, Scott Weir, Sushanta K. Banerjee

**Affiliations:** ^1^ Cancer Research Unit VA Medical Center Kansas City MO USA; ^2^ Department of Chemical Biochemical Environmental Engineering (CBEE University of Maryland Baltimore MD USA; ^3^ Department of Pathology and Laboratory Medicine University of Kansas Medical Center Kansas City KS USA; ^4^ Department of Coatings and Polymeric Materials North Dakota State University Fargo ND USA; ^5^ Department of Molecular and Integrative Physiology University of Kansas Medical Center Kansas City KS USA; ^6^ Department of Pharmacology Toxicology and Therapeutics University of Kansas Medical Center Kansas City KS USA; ^7^ Lead contact, SKB, Cancer Research Unit Kansas City MO USA; ^8^ Present address: National Institute of Biomedical Genomics Kalyani West Bengal India; ^9^ Present address: Department of Life Sciences Central University of Tamil Nadu Thiruvarur India

**Keywords:** bioavailability, breast cancer, CCN5, drug delivery, EGCG, FA‐PEG‐NPs, folic acid, nanoparticles, PCNA, TNBC

## Abstract

Epigallocatechin‐3‐gallate (EGCG) has been considered an anticancer agent despite conflicting and discrepant bioavailability views. EGCG impairs the viability and self‐renewal capacity of triple‐negative breast cancer (TNBC) cells and makes them sensitive to estrogen via activating ER‐α. Surprisingly, the mechanism of EGCG’s action on TNBC cells remains unclear. CCN5/WISP‐2 is a gatekeeper gene that regulates viability, ER‐α, and stemness in TNBC and other types of cancers. This study aimed to investigate whether EGCG (free or encapsulated in nanoparticles) interacts with the CCN5 protein by emphasizing its bioavailability and enhancing its anticancer effect. We demonstrate that EGCG activates CCN5 to inhibit in vitro cell viability through apoptosis, the sphere‐forming ability via reversing TNBC cells’ stemness, and suppressing tumor growth in vivo. Moreover, we found EGCG‐loaded nanoparticles to be functionally more active and superior in their tumor‐suppressing ability than free‐EGCG. Together, these studies identify EGCG (free or encapsulated) as a novel activator of CCN5 in TNBC cells and hold promise as a future therapeutic option for TNBC with upregulated CCN5 expression.

## Graphical abstract

CCN5 acts as a tumor suppressor/anti‐invasive protein in TNBC. Free or encapsulated EGCG treatment promotes CCN5 expression in TNBC cells to suppress the growth and progression of TNBC.

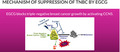

**What is already known**

- EGCG has proven to delay tumor burden.
- Mechanisms of tumor growth regulation remain unknown.

**What does this study add**

- CCN5/WISP‐2 plays a vital role in reducing cell viability and reprograming mesenchymal–epithelial transition by EGCG in TNBC.
- Free‐ and encapsulated EGCG effectively activate CCN5 and delay tumor growth in TNBC

**What is the clinical significance**

- CCN5 activation by encapsulated EGCG could be a compelling strategy to target triple‐negative breast cancer (TNBC) by enhancing bioavailability.

## 1 INTRODUCTION

Breast cancer (BC) is a genetically heterogeneous disease characterized by a mixed bag of cells.^1^ Although overall BC patients’ mortality has declined significantly, the incidence remains high. It is still the second most common cause of cancer death in women.^2^ BC is broadly classified into distinct molecular subtypes, including normal‐like, luminal A and B, HER2+, and basal‐like. The basal‐like subtype is also known as triple‐negative breast cancer (TNBC) because they lack estrogen receptor (ER), progesterone receptor (PR), and HER2.^2^ TNBC is characterized by resistance to chemotherapy, the acquisition of the stem character, and unfavorable prognoses due to its highly metastatic phenotype.^3^ TNBC patients still have minimal treatment options,^3^ and chemotherapy is currently the only treatment available for metastatic TNBC.^3^ Although checkpoint inhibitors, including programmed cell death protein 1 (PD‐1) and programmed death‐ligand 1 (PD‐L1), were found to elicit a response in TNBC in initial clinical trials,^3^ optimistic results have not yet emerged from these trials. Thus, the detection of appropriate targeted therapeutic regimens for TNBC therapy and prevention has remained an elusive challenge to many laboratories. These malignant breast tumors often consist of a small subset of the cell population, known as tumor‐initiating cells (BTICs) or cancer stem cells (BCSCs). This subpopulation of BC cells is known to be CD44 positive (CD44^+/+^) with negligible or no CD24 (CD24^low/−^) and are responsible for the acquisition of chemoresistance properties and tumor recurrence.^4^, ^5^ Hence, targeting these residual cells may act as a novel therapeutic approach to prevent tumor recurrence and improve long‐term survival in breast cancer patients.

During our program of identifying molecule(s) that could play an inhibitory role against TNBC, we found that cellular communication network factor 5 **(**CCN5, previously known as WISP‐2), a matricellular 29–35 kDa protein and a member of the CCN family of growth factors, can modulate breast cancers by imparting an inhibitory effect on tumor progression.^6^, ^7^, ^8^, ^9^ We have demonstrated that ectopic expression of CCN5 or administration of human recombinant CCN5 protein in TNBC cells resulted in suppressing tumorigenic properties and induction of growth arrest.^9^ Subsequently, the consistent role of CCN5 in tumor suppression was also reported by others.^10^, ^11^, ^12^, ^13^ CCN5 is also known to inhibit the stemness and reverse the EMT process in BC cells ^9^, ^11^ and activates estrogen receptor‐alpha (ER‐α) in TNBC cells.^14^ These recent advances, exploring the role of CCN5‐signaling in breast cancer, strongly suggest that targeting TNBC‐BCSC by activating CCN5 would be an ideal strategy to prevent breast tumors’ growth and relapse.

Epigallocatechin‐3‐gallate (EGCG), the most abundant dietary polyphenol in green tea, has been extensively studied in cancer prevention, incidence, or motility.^15^, ^16^, ^17^ EGCG has proven effective in delaying tumor incidence and significantly reducing tumor burden.^18^ Although the bioavailability of EGCG is a big concern in the clinics, multiple studies found that EGCG can activate cell‐death programs and suppress the invasiveness of TNBC both in vitro and in vivo via activation of ER‐α.^19^, ^20^, ^21^ However, the precise molecular mechanism of EGCG action on TNBC growth inhibition and suppressing invasive phenotypes is unclear. Since the recent shreds of evidence suggest that activation of CCN5 could help sensitize TNBC cells to conventional hormonal therapies through activation of ER‐α,^14^ there are possibilities that CCN5 might play a regulatory role in EGCG action against TNBC cells, particularly the BTIC subpopulation. Hence in the present study, we have explored the role of CCN5 in regulating the antitumorigenic property of EGCG against TNBC. Further, given the concern of the weak bioavailability of EGCG, we uncovered the impetuous of EGCG‐loaded nanoparticles on the reactivation of CCN5 and TNBC cells’ pathophysiology. Our studies found that free‐EGCG and encapsulated EGCG are equally effective in activating CCN5, suppressing cell growth, and oncogenic potency in vitro. However, encapsulated EGCG is significantly more effective than free‐EGCG causing tumor growth inhibition in a mice model. CCN5 reactivation by EGCG is required to render cell viability and the sphere‐forming ability of TNBC cells and sensitizes TNBC cells to EGCG if treated concurrently with CCN5 recombinant protein.

## 2 MATERIALS AND METHODS

### 2.1 Regents, chemicals, and antibodies

Epigallocatechin‐3‐gallate was purchased from Sigma Aldrich (St. Louis, MO, USA). Dulbecco's modified Eagle medium (DMEM), penicillin‐streptomycin, and trypsin–EDTA solution were purchased from Sigma (St. Louis, MO, USA), and all other chemicals were obtained from either Sigma or Fisher Scientific (Fisher Scientific, Houston, TX, USA). Fetal bovine serum (FBS) was obtained from ATCC (Manassas, VA USA). Rabbit polyclonal antibodies, such as Oct‐4 (2750), CD44 (3578), and Caspase‐3 (9665), were purchased from Cell Signaling Technologies (Beverly, MA, USA), Keratin‐19 (RB‐9021) was purchased from Thermo Scientific (Fremont, CA, USA), PCNA (sc‐7907), Bax (sc‐526), and TWIST (sc‐15393) were purchased from Santa Cruz Biotechnologies (Santa Cruz, CA, USA), and CCN5 (ab38317) was purchased from Abcam (Cambridge, MA, USA). Monoclonal mouse antibodies such as Bcl‐2 (sc‐7382) and ADH (sc‐133207) were purchased from Santa Cruz Biotechnologies, Vimentin (MS‐129) was purchased from Thermo Scientific, E‐cadherin (610404) from BD Biosciences (San Jose, CA, USA), and GAPDH from Applied Biosystems (Foster City, CA, USA). The dilution of the antibody was made as per the manufacturer's recommendations, ~1:500 to 1:1000. All chemicals for the generation of nanoparticles were purchased from Sigma‐Aldrich, solvents were purchased from EMD Millipore, and used without further purification. PEG derivatives were obtained from Laysan Bio. Glassware was washed using aqua regia and dried in an oven overnight before use.

### 2.2 Cell lines and culture condition

Human breast cancer cell lines, such as MCF‐7 (ER‐α positive), MDA‐MB‐231, and HCC‐70 (TNBC), and mouse 4T1 TNBC cell line and Panc‐1 (human pancreatic adenocarcinoma cell line) were purchased from American Type Culture Collections (ATCC, Manassas, VA) and cultured in Dulbecco's Modified Eagle Medium (Sigma, MO) containing 10% FBS (ATCC) at 37°C in a humidified chamber. Cell lines are maintained at ≤18 passage from receipt and were characterized using short tandem repeat analysis before initial culture. Besides, cell lines were routinely checked for mycoplasma contamination.

### 2.3 Cell viability assay

Antiproliferative effects of EGCG (0–100 µM) on various cancer cells were determined by crystal violet assay following the protocol published previously.^22^ Cells were treated with EGCG for 72 hours and, after treatment, subjected to crystal violet staining for 10 min. The resultant crystal violet complex was then dissolved in 10% acetic acid, and the absorbance was measured at 600 nm, using a SpectraMax‐340 microplate reader (Molecular Devices, Sunnyvale, CA). Cell viability was calculated using the following mathematical expression:

(1) $$\%\text{inhibition}=\left[1‐\text{At}/\text{As}\right]\times 100\%$$

[At and As indicated the absorbance of the test substances and solvent control, respectively^23^].

### 2.4 Apoptosis assay

MDA‐MB‐231 cells were treated with different doses of EGCG (0–75 µM) for 72 h, and apoptosis was determined by annexin‐V‐FITC/propidium iodide (PI) double staining method, using flow cytometry.^24^ Results were obtained on FACSCalibur (BD, CA) using the Cell Quest software (BD, San Jose, CA, USA).^25^

### 2.5 Anchorage‐dependent growth (ADG) assay

Anchorage‐dependent growth (ADG) or colony‐formation assay for EGCG‐treated and untreated cells was performed by following the previously published protocol.^22^ To determine the colony‐forming ability of TNBC cells, MDA‐MB‐231 cells were treated with EGCG (0–75 µM) for 72 hours and seeded at a density of 2 cells/μl. EGCG‐treated or untreated MDA‐MB‐231 cells were distributed in two treatment groups. In one group, pretreated cells were subjected to colony formation for 7 days (pretreatment group‐T1) in the absence of EGCG. In contrast, in another group, pretreated cells were seeded for colony formation and subjected to EGCG treatment for the remaining 6 days (posttreatment group‐T2). Colonies were then stained with crystal violet, and the number of colonies was determined using the Colony Doc‐It Image station (UVP, Upland, CA**)**.

### 2.6 Mammosphere assay

BC cells’ mammosphere‐forming ability was determined using the commercially available MammoCult^TM^ media (Stemcell Technologies, Vancouver, Canada). Complete media for mammosphere culture were prepared according to the manufacturer's protocol by supplementing MammoCult^TM^ basal medium with freshly prepared hydrocortisone (0.48 μg/ml) and heparin (4 μg/ml). Briefly, MDA‐MB‐231 cells were treated with EGCG treatment (75 µM) in the presence or absence of CCN5 recombinant protein (hrCCN5, 100 ng/ml) or CCN5 neutralizing antibody (CCN5^ab^, 300 ng/ml) or left untreated (control). Cells were then seeded at a density of (2 cells/μl) and propagated in low attachment dishes for 8–10 days. Sphere formation was initiated after 3 days of culture. A steady increase or decrease in the size and number of mammospheres was observed in control and treated MDA‐MB‐231 cells. At the end of the experiments, the size and number of the mammospheres were measured.

### 2.7 Real‐time PCR

The qRT‐PCR mixture was prepared using SYBR green master mix (Applied Biosystems). Master mix and cDNA were combined in a 48‐well plate, and the samples were run in an Applied Biosystem StepOne Real‐time PCR machine. The CCN5 and GAPDH primers were used as listed in Table S1. The relative changes of gene expression were calculated using the following formula: fold change in gene expression, 2^−ΔΔCt^ = 2^−{ΔCt (treated samples) − ΔCt (untreated control sample)^, where ΔCt = Ct (CCN5) ‐ Ct (GAPDH) and Ct represents threshold cycle number.

### 2.8 Luciferase activity assay for CCN5 promoter

MDA‐MB‐231 and MCF‐7 cells were seeded in 96‐well tissue culture plates and grown to ~70% confluency. Cells were then transfected with pLight‐Switch_Prom reporter plasmid containing CCN5 promoter (Switchgear Genomics, Carlsbad, CA) and the corresponding empty promoter using FuGENE‐HD transfection reagent (Promega, Madison, WI).^9^ Transfected cells were then treated with EGCG for 48 h, and luciferase assay was performed using the Light‐Switch Luciferase Assay Kit (Switchgear Genomics, CA), following the manufacturer's protocol. Luminescence was monitored by GloMax TM Luminometer (Promega, Madison, WI).

### 2.9 Immunohistochemistry

All immunohistochemistry experiments were performed using the Histostain‐Plus IHC Kit (Life Technologies) following the previously published protocol.^9^ Briefly, after microwave treatment of deparaffinized tissue sections in citrate buffer and endogenous peroxide blocking, the sections were incubated in a specific antibody solution overnight at 4°C in a moist chamber. The immune reactivity was detected by DAB (3,3′‐Diaminobenzidine), and the sections were counterstained with hematoxylin.

### 2.10 Western blot analysis

Western blot analysis was performed to determine the expression levels of the apoptotic proteins, EMT‐related proteins, and CCN5 in untreated and EGCG‐treated BC cell lines using our previous method.^26^ Chemiluminescence signal was detected by Super Signal ULTRA Chemiluminescent Substrate (Pierce, Rockford, IL, USA), using ID Image Analysis Software Version 3.6 (Eastman Kodak Company, Rochester, NY, USA). Uncropped images of Western blot using these studies are shown in Figure S1.

### 2.11 Generation and characterization of EGCG nanoparticles (NPs)

**FIGURE 1:**
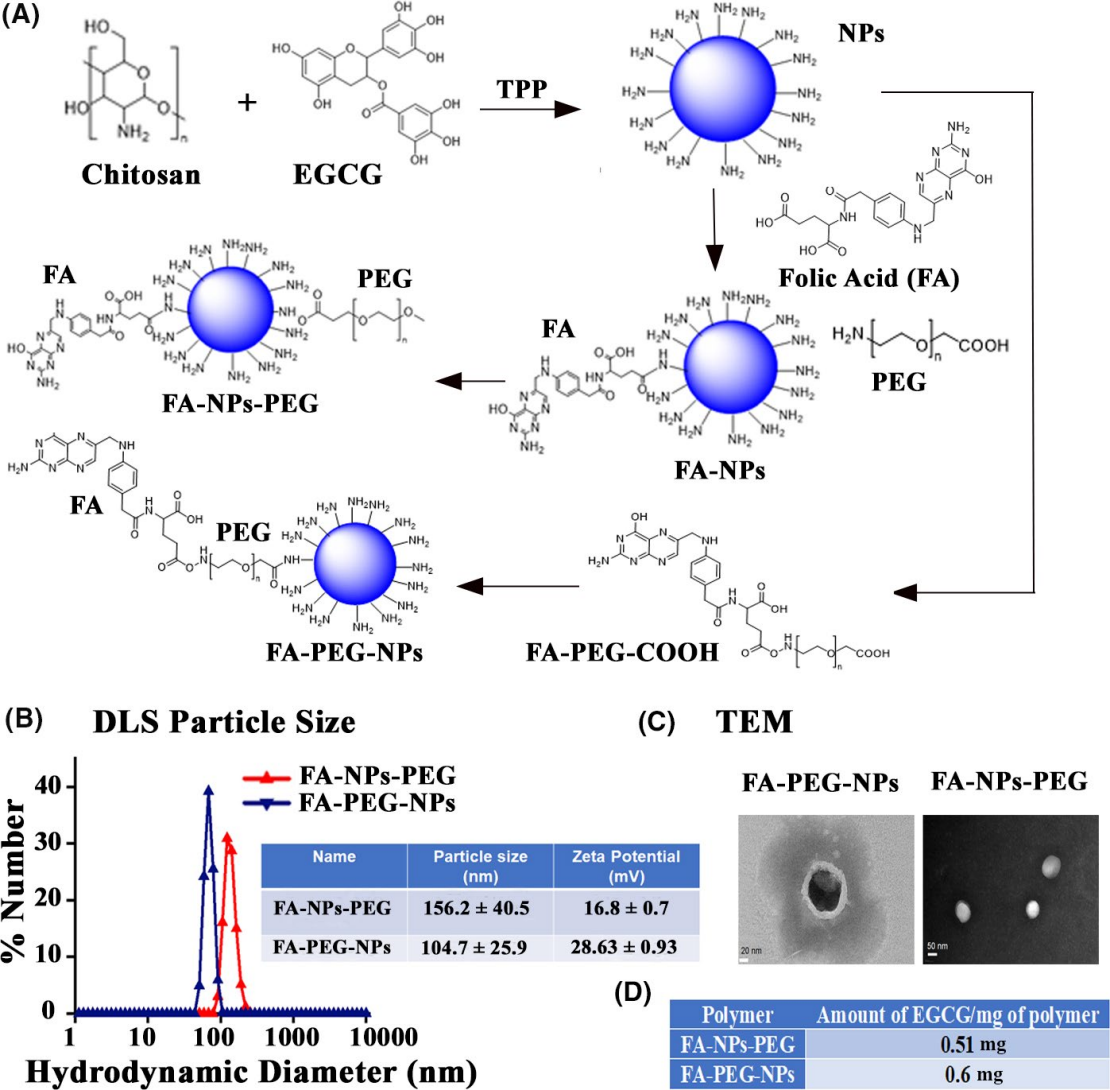
Diagrammatic illustration of spatio‐selective activation and synthetic route of EGCG‐loaded tumor cell‐targeted nanoparticles. (A) The schematic illustration of the synthetic route toward the preparation of nanoparticles. (B) Plot and table of hydrodynamic diameters of EGCG containing nanoparticles. (C) Images of nanoparticles obtained using transmission electron microscopy (TEM). (D) Table showing the amount of drug in each nanoparticle formulation

The EGCG‐NPs were prepared and characterized as the protocol described earlier.^27^ Two types of nanoparticles, viz., FA‐NPs‐PEG and FA‐PEG‐NPs, were prepared as described in Figure 1. FA‐NPs‐PEG was prepared by crosslinking chitosan and EGCG using tripolyphosphate (TPP), in which EGCG was encapsulated. The next step involved the addition of folic acid (FA) via 1‐Ethyl‐3‐(3‐dimethylaminopropyl)carbodiimide (EDC) coupling between the NH_2_ groups of chitosan and ‐COOH groups of FA. Polyethylene glycol (PEG) was then conjugated to this system via ‐CONH‐ bonds between ester group (‐COOR) of succinimidyl ester of PEG propionic acid (mPEG‐SPA) and ‐NH2 group of chitosan. For the synthesis of FA‐PEG‐NPs, FA was first activated using N‐Hydroxysuccinimide (NHS) and N, N’‐dicyclohexylcarbodiimide (DCC) in DMSO, and it was then added to a PEG derivative (NH2‐PEG‐COOH) using carbodiimide reaction. Then, the ‐COOH groups of FA‐PEG‐COOH were further activated using EDC and NHS to generate the NHS derivative. The resultant derivative was then grafted onto chitosan and further crosslinked with EGCG using TPP. The particle size and morphology were obtained by dynamic light scattering (DLS) (Figure 1B) and transmission electron microscopy (TEM) (Figure 1C), respectively. Drug concentration was measured using UV‐Vis absorbance spectroscopy.

### 2.12 Cellular uptake studies

Cellular uptake of nanoparticles was determined as described previously.^27^ Briefly, TNBC cells were cultured with a density of 20,000 cells/well in six‐well plates for 2 days to achieve 70% confluent monolayer. Subsequently, Alexa Fluor 647 (AF‐647)‐labeled nanoparticle suspensions (80 μl or equivalent amount containing 75 μM EGCG) were added to cells. Following the treatment at various time points (1, 5, and 24 hours), fluorescence emission intensity was visualized and quantified using a Nikon Eclipse 90i microscope equipped with imaging software. The fluorescence intensity was quantified using NIS Elements BR Software and verified by the ImageJ 1.45s Software (NIH, Bethesda, MD).

### 2.13 Tumor xenografts in Athymic Female Nude Mice

Six‐ to 8‐week‐old athymic female nude mice were obtained from Jackson Laboratory and were used for tumor development. According to standard guidelines of the American Association for the Accreditation of Laboratory Animal Care, all mice were maintained with the approval of the Institutional Animal Care and Use Committee of the Kansas City VAMC. Cultured MDA‐MB‐231 cells (1 × 10^6^) were suspended in 0.1 ml PBS and 0.1 ml Matrigel (BD, San Jose, CA, USA), and the mixture was injected subcutaneously into the right hind leg of each mouse for the development of the tumor. After developing the palpable tumor (~50 mm^3^), mice were divided into control and treated groups (n = 4). In the treatment group, mice were orally administered with EGCG (100 mg/Kg)/day for 3 weeks. For nanoparticle experiments, tumor‐bearing mice were injected (intraperitoneal) EGCG‐FA‐PEG‐NPs (25 μg/mice/100 μl solution/day) or free FA‐PEG‐NPs for 2 weeks. We considered 2 weeks to compare the difference between free and encapsulated EGCG. When tumors reached a volume of 400 mm^3^, mice were euthanized, and tumors were collected for further investigations. Tumor volume was measured and calculated every 2 days by the formula 0.5 × *w^2^* × *l* (where *w* = width and *l* = length) using Studylog’ software.

### 2.14 Statistical analysis

The data arrangement, organization, and statistical analysis were performed as per Michel et al.^28^ All data are presented as the mean ± SD of “*n*” independent measurements, as indicated in the corresponding figure legends. Statistical comparisons between treated and untreated control groups were calculated by Student's *t* tests using GraphPad Prism 6, and multiple groups were determined by ANOVA test. A value of *p* < 0.05 was considered significant.

### 2.15 Nomenclature of targets and ligands

Key protein targets and ligands in this article are hyperlinked to corresponding entries.

In http://www.guidetopharmacology.org, the common portal for data from the IUPHAR/BPS Guide to PHARMACOLOGY (Harding et al., 2018),^29^ and are permanently archived in the Concise Guide to PHARMACOLOGY 2019/20 (Alexander et al., 2019).^30^

## 3 RESULTS

### 3.1 EGCG transcriptionally activates CCN5 in breast cancer cells

**FIGURE 2:**
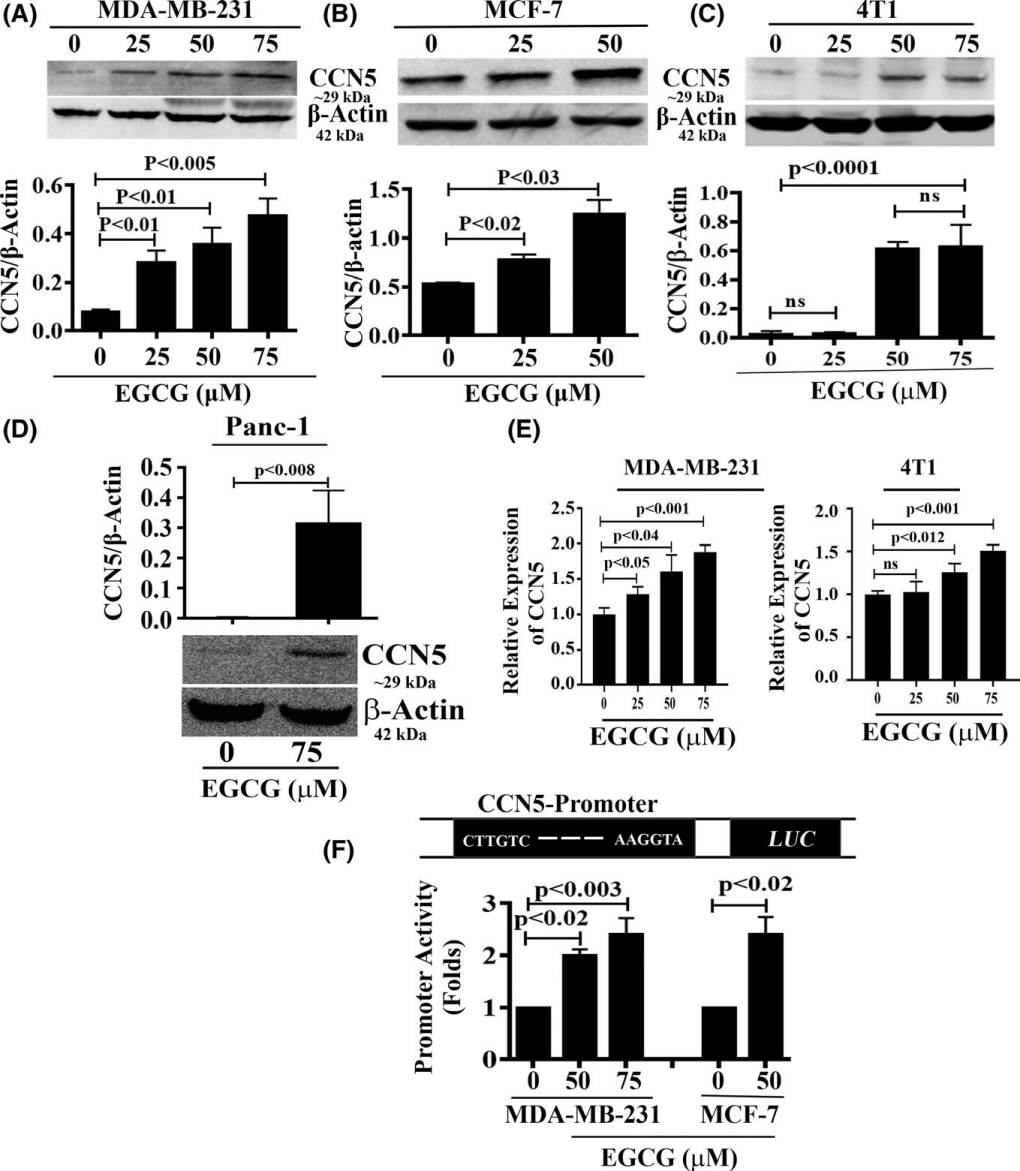
EGCG reactivates CCN5 through transcriptional activation. (A‐C). Immunoblot analysis and quantification of CCN5 in lysates of untreated and different doses of EGCG‐treated MDA‐MB‐231, MCF‐7, and 4T1 TNBC cell lines. P‐value determined by Student's *t* test, data are mean ± SD when n = 3. (D) Immunoblot analysis and quantification of CCN5 in lysates of untreated and EGCG‐treated Panc‐1 pancreatic cancer cell line. P‐value determined by Student's t‐test, data are mean ± SD when n = 3. (E) Quantification of relative expression of CCN5 mRNA in EGCG‐treated MDA‐MB‐231 and 4T1 cell extracts using qRTPCR. P‐value determined by Student's *t* test, data are mean ± SD when n = 8. (F) Dose‐dependent induction of the CCN5 promoter constructs by EGCG in MDA‐MB‐231 and MCF‐7 cell lines. CCN5 promoter‐luciferase was performed as described under the Method section. P‐value determined by Student's *t* test, data are mean ± SD when n = 3

Both CCN5 and EGCG restore ER‐α expression and activity in TNBC cells.^14^, ^19^, ^21^ In this study, we investigated whether EGCG can induce or enhance CCN5 expression in TNBC cell lines. We treated different cell lines (i.e., MCF‐7, MDA‐MB‐231, and 4T1) with different doses of EGCG, and the expression level of CCN5 protein was monitored using Western blot analysis. We found a dose‐dependent increase of CCN5 expression in these cell lines after 48 hours of treatment (Figure 2A‐C). EGCG with a dose of 50 μM significantly upregulated CCN5 expression in all three cell lines. The promoting effect of EGCG with a 25 μM dose was only detected in MDA‐MB‐231 and MCF‐7 cell lines.

Previously, we have reported that similar to TNBC cell lines, induced overexpression of CCN5 in pancreatic ductal adenocarcinoma (PDAC) cells promotes mesenchymal–epithelial transition (MET) and weakens the steaminess of these aggressive cells^31^. Thus, we tested whether EGCG treatment effectively upregulates CCN5 expression in Panc‐1 cells, an aggressive PDAC cell line. We found that CCN5 protein level significantly increased in Panc‐1 cells following EGCG treatment for 48 hours (Figure 2D).

Next, we determined whether EGCG transcriptionally regulates CCN5 expression. To test the premise, we first examined the effect of different concentrations of EGCG on CCN5 mRNA expression in MDA‐MB‐231 and 4T1 cell lines using qPCR analysis. The studies showed a dose‐dependent effect of EGCG on mRNA expression in these cells (Figure 2E).

Finally, a luciferase assay was performed to measure the promoter activity of CCN5 after transfecting MCF‐7 and MDA‐MB‐231 cells with LightSwitch_Prom reporter plasmid containing the CCN5 promoter. We found that EGCG significantly increased CCN5 promoter activity in a dose‐dependent fashion compared to the untreated control cells (Figure 2F). In the presence of 50 µM and 75 µM EGCG, CCN5 promoter activity was increased by 2‐fold and 2.4‐fold, respectively, in MDA‐MB‐231 cells. In EGCG‐treated MCF‐7 cells, CCN5 promoter activity was increased by 2.4‐fold at a dose of 50 μM compared to untreated control. These results indicate that EGCG treatment of breast cancer cells was resulting in the transcriptional activation of CCN5.

### 3.2 EGCG decreases cell viability through apoptosis in BC cells via upregulation of CCN5

**FIGURE 3:**
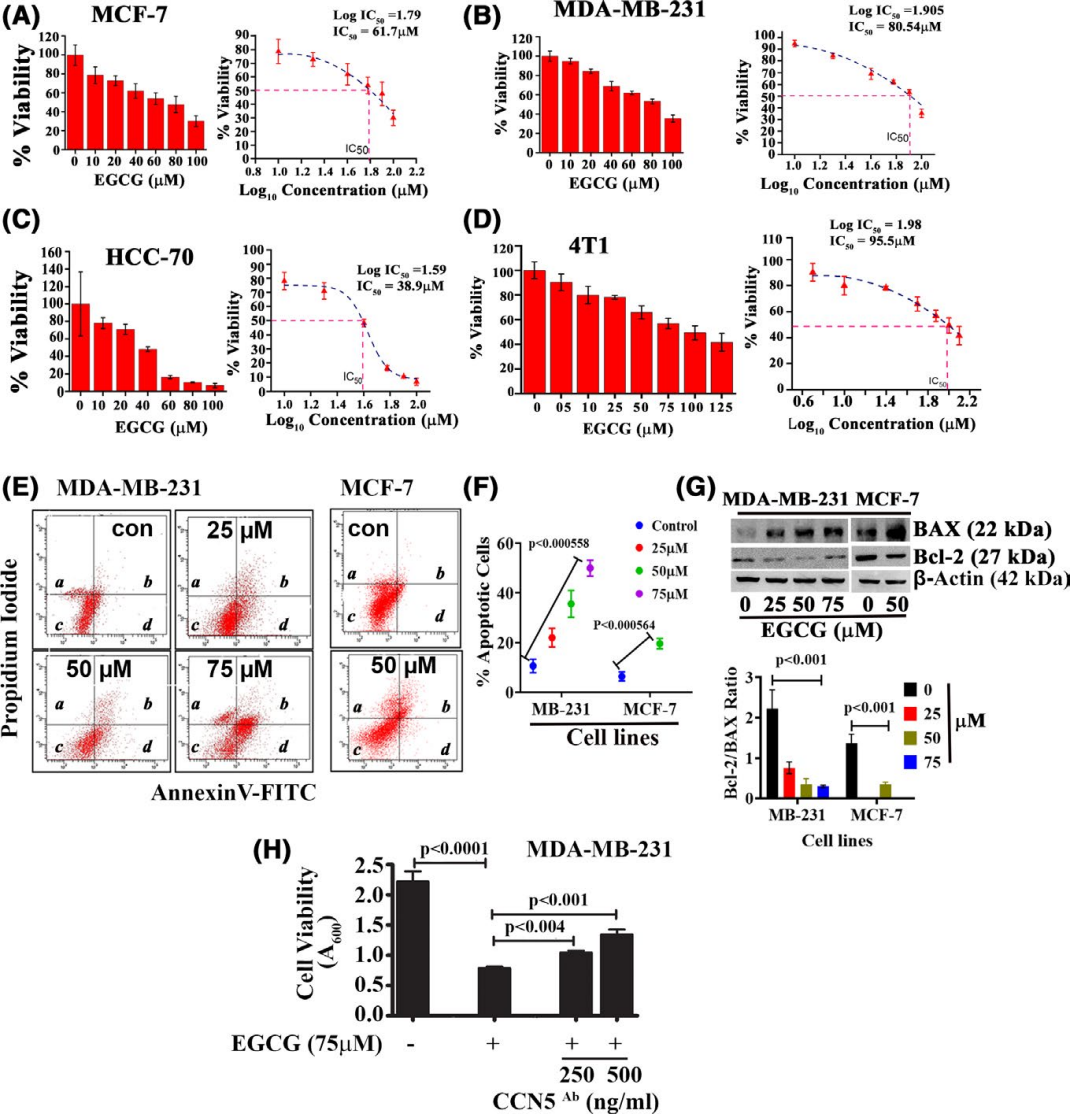
EGCG reduces cell viability via apoptosis. (A‐D) Dose‐dependent effect of EGCG on cell viability and defined the IC_50_ in MCF‐7 and TNBC cell lines. P‐value determined by Student's *t* test, data are mean ± SD when n = 3. (E‐F) Detection and quantification of apoptotic cells using propidium iodide‐flow cytometry. The graph shows the mean ± SD of three independent experiments. (G) EGCG‐treated MDA‐MB‐231 and MCF‐7 cell lysates were analyzed by immunoblot to detect BAX and Bcl‐2 proteins. The graph shows the mean ± SD of three independent experiments. (H) Detection of cell viability in MDA‐MB‐231 cells treated with EGCG in the presence or absence of CCN5 neutralizing antibody

The cell viability studies demonstrate a dose‐dependent effect of EGCG on cell killing in four BC cell lines (Figure 3A‐D). These include MCF‐7, MDA‐MB‐231, HCC‐70, and 4T1. The respective IC_50_ values obtained for MCF‐7, MDA‐MB‐231, HCC‐70, and 4T1 cells were 61.7 µM, 80.54 µM, 38.9 µM, and 95.5 μM, respectively.

To determine whether EGCG‐induced loss of cell viability is due to apoptosis, annexin V‐FITC/PI double staining was performed. We found that EGCG enhances apoptosis in both MDA‐MB‐231 and MCF‐7 cells in a dose‐dependent fashion (Figure 3E and F) via shifting the Bcl‐2/BAX (antiapoptotic/apoptotic protein) ratio toward apoptosis (Figure 3G).

Although EGCG upregulates CCN5 expression in breast cancer cells, the link between CCN5 activation and EGCG‐mediated suppression of TNBC cell viability is unknown. Our current studies found that EGCG‐induced cell death can be rescued by the concomitant treatment of CCN5 antibody in a dose‐dependent fashion (Figure 3H).

### 3.3 CCN5 recombinant protein therapy synergizes EGCG’s sensitivity on cell viability

**FIGURE 4:**
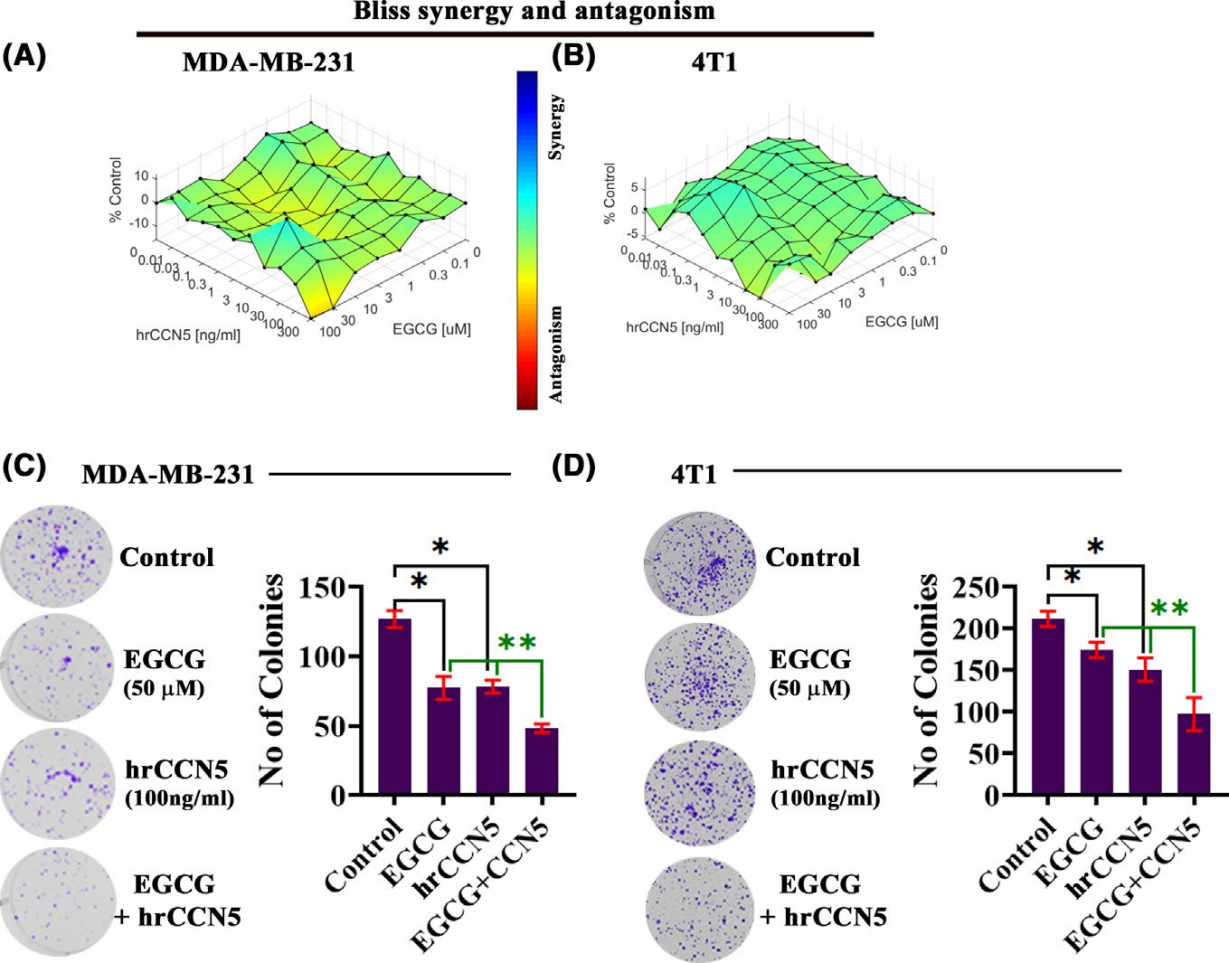
Effect of EGCG and human recombinant CCN5 (hrCCN5) protein on TNBC cell viability and the synergistic cytotoxic activity of combined EGCG and hrCCN5. (A‐B) The dose‐dependent synergistic cytotoxic activity of combined hrCCN5 and EGCG on TNBC cell lines. (C‐D) Combination treatment of hrCCN5 and EGCG on colony‐forming ability of TNBC cells reveals synergy. The graphs show the mean ± SD of three independent experiments

We have previously shown that human recombinant CCN5 (hrCCN5) protein treatment slightly but significantly suppresses TNBC cell viability. Thus, we investigate whether hrCCN5 protein treatment enhances EGCG sensitivity on TNBC cell lines’ viability. MDA‐MB‐231 and 4T1 cells were exposed to EGCG alone or in a combination of EGCG and hrCCN5 protein in various concentrations for 48 hours. The synergy (additive or super‐additive) effect of hrCCN5 and EGCG was evaluated using the Loewe model,^32^, ^33^ and the results are illustrated in Figure 4. Loewe analysis was conducted using the Combenefit® software.^33^ We found that the combination treatment of hrCCN5 protein and EGCG shows dose‐dependent multiple additives and super‐additive effects on MDA‐MB‐231 and 4T1 cell viability. The clonogenic assay results further supported the synergetic effect of the combination treatment of EGCG and hrCCN5 (Figure 4C and D).

### 3.4 EGCG inhibits the sphere‐forming ability of TNBC cells through upregulation of CCN5

Cancer stem cells, also known as TICs, are responsible for metastasis, tumor relapse, and acquisition of chemoresistance properties.^4^, ^5^, ^34^ One of the critical hallmarks of CSCs is their ability to grow anchorage independently under serum‐free culture conditions, thus resulting in the formation of tumorspheres.^35^, ^36^, ^37^ The basal‐like triple‐negative MDA‐MB‐231 cells form mammospheres when propagated under non‐differentiating culture conditions.^38^, ^39^ Therefore, in this study, we first determined the effect of EGCG on the sphere‐forming ability of MDA‐MB‐231 cells and stem cell markers. We found that sphere formation was initiated in both untreated and treated sets after 3 days of culture, and a steady increase in both the size and number of mammospheres was observed in control cells.

**FIGURE 5:**
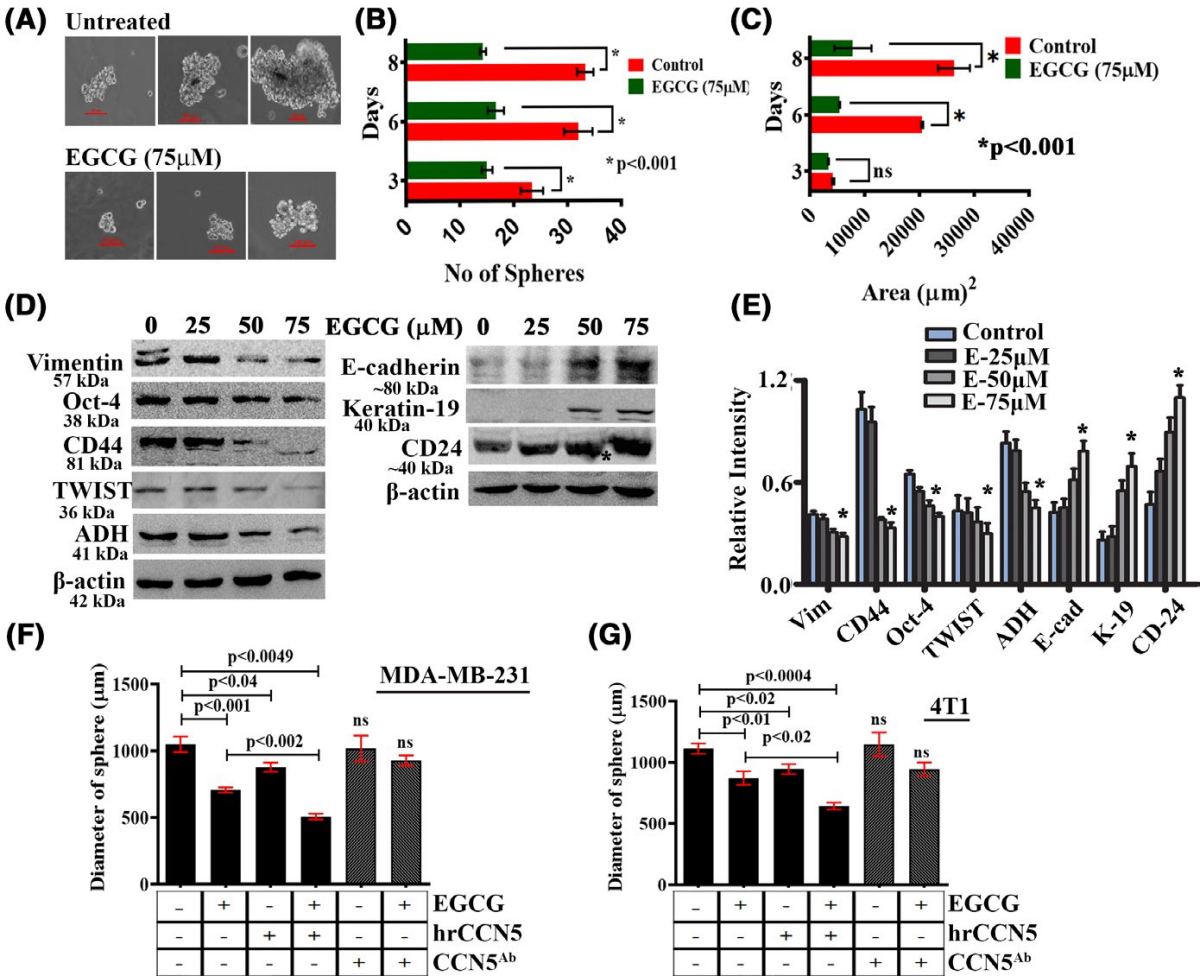
Suppression of mammosphere‐forming ability of TNBC cells by EGCG was enhanced by hrCCN5 protein and rescued by CCN5 antibody treatment. (A‐C) Effect of EGCG on the sphere‐forming ability of MDA‐MB‐231 cells. The graphs show the mean ± SD of five independent experiments. (D‐E) Immunoblot analysis and quantification of mesenchymal/stemness (left) and epithelial (right) protein markers in lysates of untreated and EGCG‐treated MDA‐MB‐231 cells. The graph shows the mean ±SD of five independent experiments. F‐G. Sphere‐forming ability of MDA‐MB‐231 and 4T1 cells were measured following treatment with EGCG alone or a combination of hrCCN5 protein or CCN5 antibody (CCN5^Ab^). The graphs show the mean ± SD of five independent experiments

In contrast, the growth rate and the number of spheroids have significantly reduced in treated cells in a time‐dependent manner (Figure 5A‐C). Hence, these results suggest that EGCG targets breast CSCs and abrogates their self‐renewal properties. To expand upon these results, we examined the expressions of epithelial–mesenchymal (EMT) markers/stemness markers in MDA‐MB‐231 cells. We found that the expressions of several mesenchymal markers such as Vimentin, Oct‐4, CD44, TWIST, and ADH were drastically reduced, while some epithelial markers markedly elevated in EGCG‐treated MDA‐MB‐231 cells (Figure 5D‐E). We then determined whether CCN5 plays any role in the blockage of sphere formation of TNBC cells by EGCG, and to test this, MDA‐MB‐231 and 4T1 cells were treated with EGCG in the presence or absence of hrCCN5 or CCN5 antibody (CCN5^Ab^) for 48 h. The untreated and treated cells were reseeded for mammosphere formation. We found that hrCCN5 protein‐treated cells exhibited greater sensitivity to EGCG than EGCG‐alone treated, while CCN5^Ab^‐treated cells were less sensitive to EGCG (Figure 5F‐G). These findings provide a mechanistic basis for EGCG therapy and indicate that CCN5 is the target for activation of EGCG to exert its anti‐EMT effect.

### 3.5 Effect of EGCG‐loaded nanoparticles on CCN5 expression in TNBC cells

**FIGURE 6:**
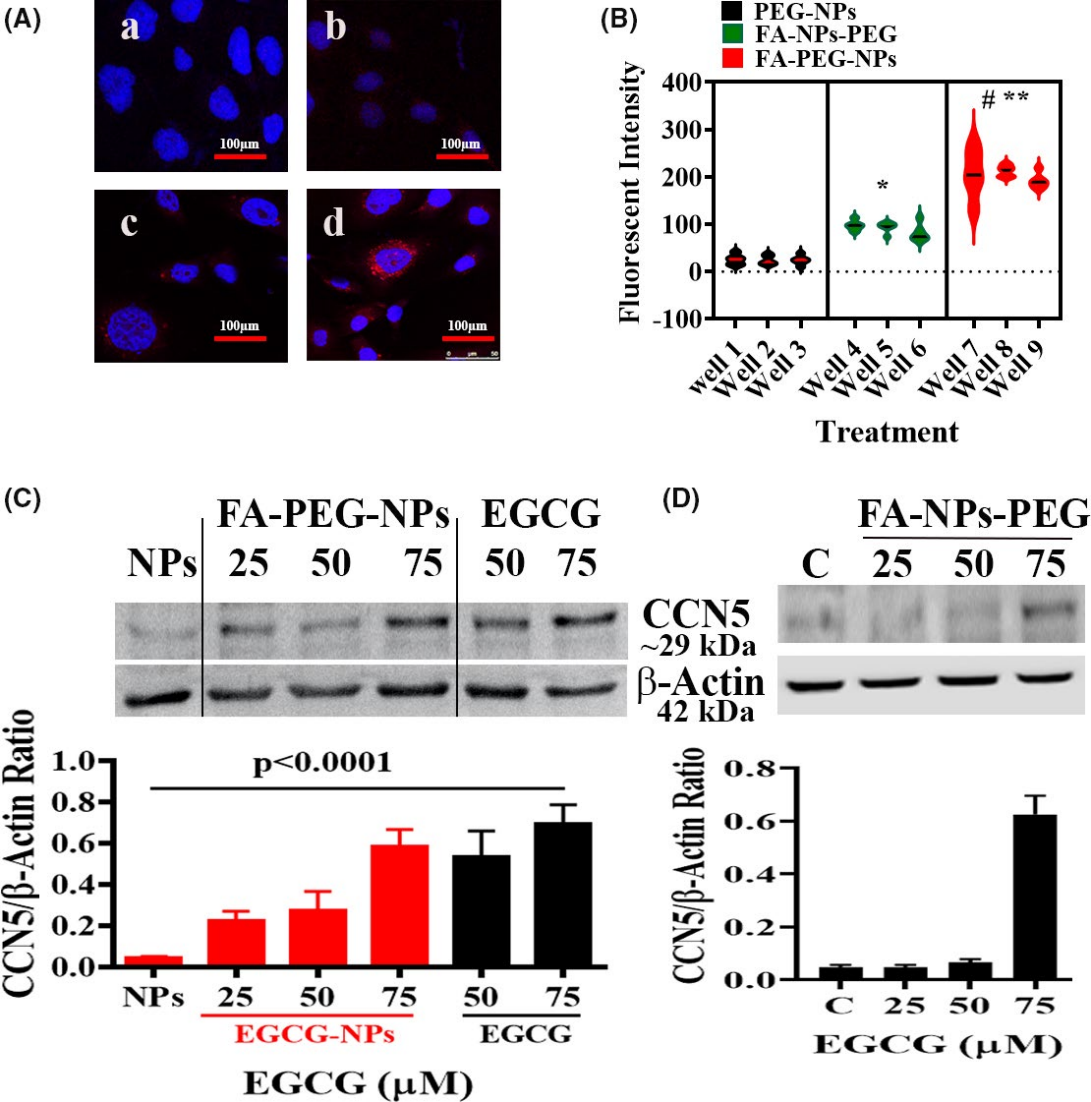
Differential effect of EGCG‐loaded structurally different nanoparticles on CCN5 reactivation in MDA‐MB‐231 cells. (A‐B) Confocal images of MDA‐MB‐231 cells showing cellular uptake of fluorescently labeled EGCG‐loaded NPs with the different chemical structures after 24 h of incubation. Detailed protocols are described in the Method section. a, nanoparticle without fluorescently tagged, b, PEG‐NPs, c, FA‐NPs‐PEG, and d, FA‐PEG‐NPs. (C‐D) Immunoblot analysis of CCN5 expression in the lysates of MDA‐MB‐231 cells treated with free‐ or encapsulated EGCG. β‐Actin was used as a loading control. The graph shows the mean ± SD of three independent experiments

Although EGCG exhibits numerous promising health‐promoting impacts in several in vitro and in vivo studies, weak bioavailability is a critical issue observed through clinical trials.^40^ The nanoformulations of supplements provide a new dimension in improving bioavailability, protect active ingredients against degradation, or reduce side effects.^27^, ^41^ Given the importance of nanoformulations, in this study, we determined the efficacy of EGCG‐loaded nanoparticles (EGCG‐NPs),^27^ as described in Figure 1, on CCN5 expression in TNBC cells in vitro. Previous studies have shown that the cellular uptake of FA‐NPs, FA‐NPs‐PEG, and FA‐PEG‐NPs was considerably higher than NPs and PEG‐NPs and suggested that this could be the presence of FA, which binds with the cancer cells having FA receptor.^27^ Therefore, in this study, we first established the targeting ability of nanoparticles, and to do so, we determined the cellular uptake of EGCG‐encapsulated nanoparticles. As shown in Figure 6A‐B, the cellular uptake of nanoparticles, based on fluorescence intensities, was significantly higher in cells treated with FA‐PEG‐NPs as compared to FA‐NPs‐PEG‐ and PEG‐NPs‐treated cells. However, a significant increase in cellular uptake of FA‐NPs‐PEG was also observed when compared with PEG‐NPs‐treated cells. The cellular uptake data suggest that FA's spatial arrangement within the chemical constructs is critical to portend the nanoparticles’ sensitivity.

Based on the above cellular uptake results, we anticipated that EGCG containing FA‐PEG‐NPs could be more effective than EGCG containing FA‐NPs‐PEG in cancer cells’ physiology. Thus, we first investigated the effect of FA‐NPs‐PEG and FA‐PEG‐NPs on CCN5 expression in TNBC cells. We found that FA‐PEG‐NPs significantly upregulated CCN5 protein expression in a dose‐dependent fashion compared to drug‐free NPs (Figure 6C). On the other hand, the effect of FA‐NPs‐PEG on CCN5 was weak compared to FA‐PEG‐NPs (Figure 6D). The dose‐dependent studies revealed that FA‐NPs‐PEG upregulated CCN5 at the dose of 75 μM, while the effect of FA‐PEG‐NPs on CCN5 expression was first detected at the dose of 25 μM. In conclusion, EGCG containing FA‐PEG‐NPs was found to be functionally superior to FA‐NPs‐PEG in upregulating CCN5 in TNBC cells.

### 3.6 Effect of EGCG‐loaded nanoparticles on in vitro cell viability and sphere‐forming ability of TNBC cells

**FIGURE 7:**
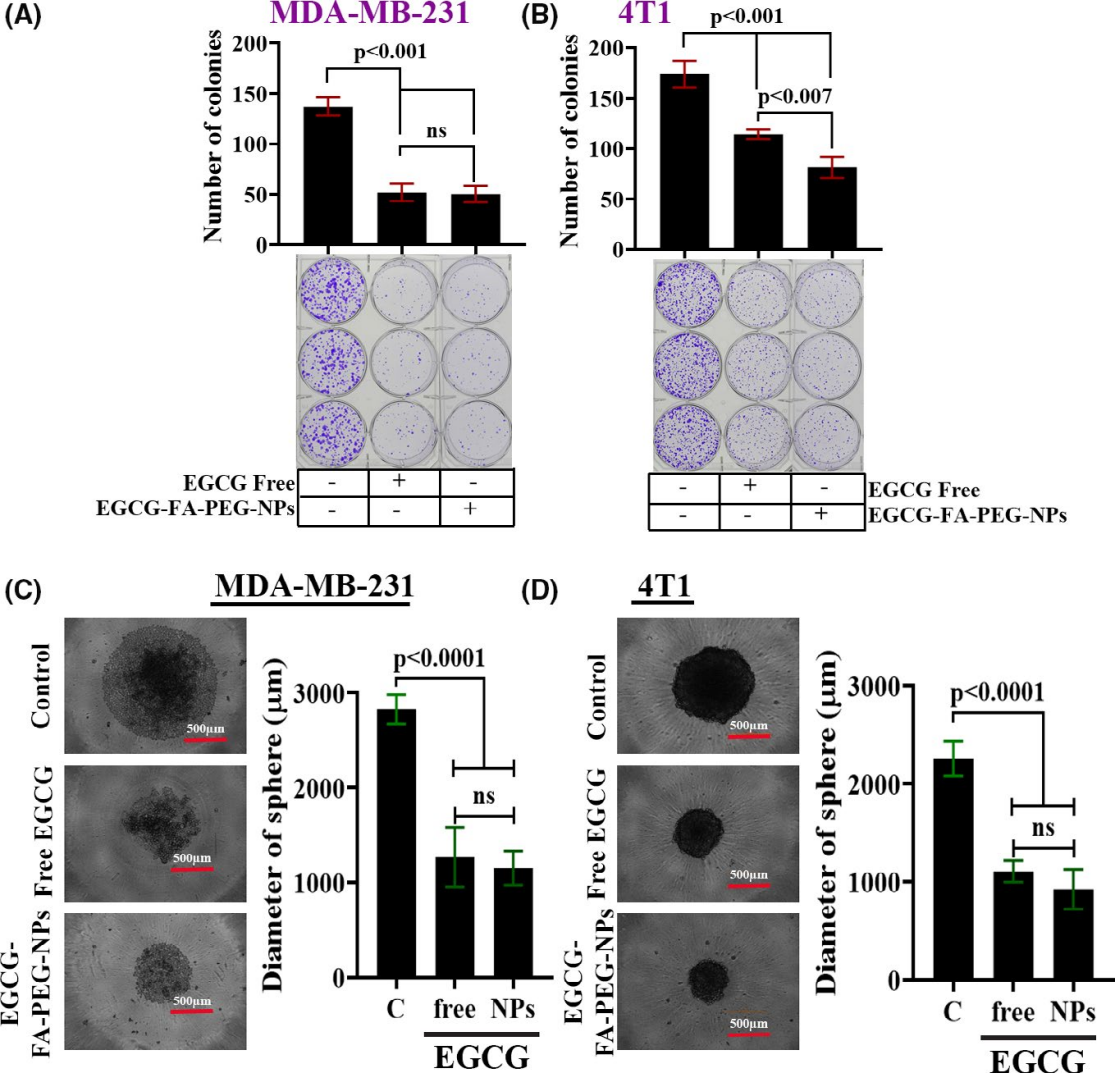
EGCG‐loaded FA‐PEG‐NPs is equally effective as free‐EGCG in inhibiting colony‐ and sphere‐forming ability of TNBC cells. (A‐B) The colony‐forming ability of free‐EGCG and EGCG‐loaded nanoparticles treated TNBC cells as determined using anchorage‐dependent growth (ADG) assay. The graph shows the mean ± SD from triplicate measurements, ns, non‐significant. (C‐D) The sphere‐forming ability of free‐EGCG and EGCG‐loaded nanoparticles treated TNBC cells as determined using the mammosphere assay (C). The graph (D) shows the mean ± SD from triplicate measurements, ns, non‐significant

To gain some insight into EGCG‐loaded nanoparticles’ potential, we next examined the capacity of EGCG containing FA‐PEG‐NPs to inhibit TNBC cell viability and sphere‐forming ability. We treated MDA‐MB‐231 cells and 4T1 cells with free‐EGCG, EGCG‐encapsulated FA‐PEG‐NPs, or left untreated for 48 hours. The cells were then reseeded for ADG and sphere‐formation of MDA‐MB‐231 cells and 4T1 cells. We observed that both free and encapsulated EGCG‐treated cells lost their ability to form colonies (Figure 7A‐B) and mammospheres (Figure 7C and D) compared to untreated cells. Interestingly, we found that the impact of EGCG‐NPs on inhibition of colony formation was significantly higher in 4T1 cells. Collectively, these in vitro studies suggest functional equivalences of EGCG‐loaded nanoparticles and free‐EGCG.

### 3.7 Effect of free‐ or encapsulated EGCG on tumor propagation in MDA‐MB‐231‐mouse xenografts

**FIGURE 8:**
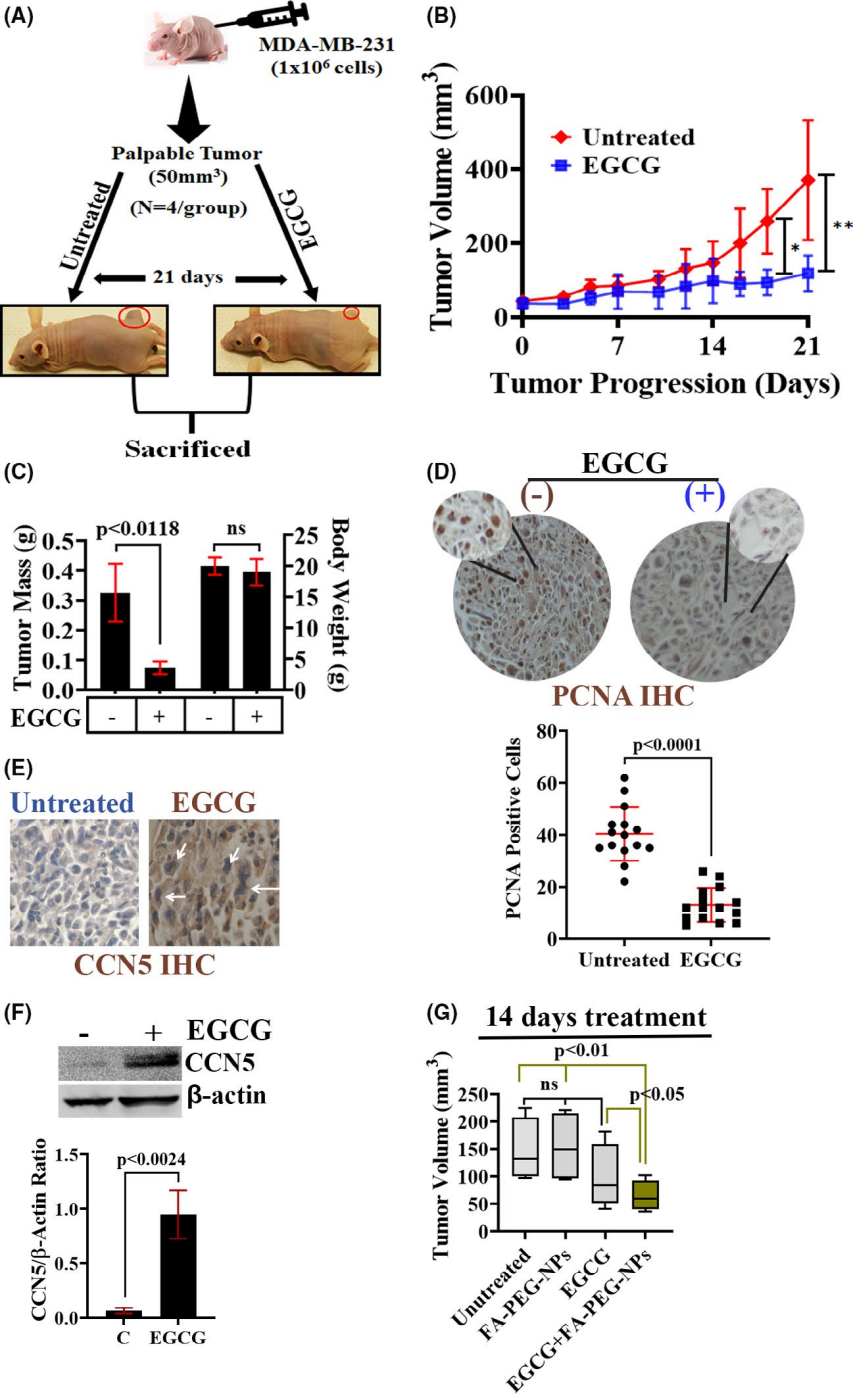
Inhibition of tumor growth in a xenograft model by EGCG correlates with CCN5 expression level in tumor tissue. (A‐C) Scheme of the EGCG treatment strategy (A). The figure illustrates the outcome of the effect of EGCG on the MDA‐MB‐231‐tumor xenograft mouse model (A). Quantification of tumor volume (B) and tumor mass (C) in the MDA‐MB‐231‐xenograft mouse model. Data represent error bar mean ± SD, n = 4 animal/group, ns = non‐significant. (D) IHC staining of PCNA and quantification of PCNA‐positive cells in EGCG‐treated and untreated MDA‐MB‐231‐xenograft tumor sections. Data represent error bar mean ± SD, n = 4 animal/group. (E) IHC staining of CCN5 in EGCG‐treated and untreated MDA‐MB‐231‐xenograft tumor sections. (F) Western blot analysis of CCN5 in lysates of untreated and EGCG‐treated MDA‐MB‐231‐tumor xenograft samples. Data represent error bar mean ± SD, n = 4 animals/group. P‐value determined by one‐way ANOVA and Student's *t* test, data represent error bar mean ± SD, n = 4 animals/group. (G) Quantification of tumor volume in free‐EGCG and EGCG‐loaded nanoparticles treated (14 days) tumor xenograft mouse model. Data represent error bar mean ± SD, n = 4 animal/group

To better reflect the clinical context, we verified the effectiveness of free‐ and nanoparticle‐encapsulated EGCG on tumor growth and progression under in vivo setting. First, we generated subcutaneous TNBC tumors by injecting MDA‐MB‐231 cells in athymic (nude) mice. The palpable tumors were detected within 7 days of injection, and the tumor‐bearing mice were treated with EGCG at a dose of 100 mg/kg body weight. Free‐EGCG was administered orally by gavage for ~21 days. EGCG treatment halted tumor progression via impairing cell growth as the number of PCNA‐positive cells was reduced significantly in EGCG‐treated tumors without a toxic impact on tumor‐bearing mice's body weight (Figure 8A‐D). The significant inhibition of tumor growth was first detected on day 18 of the treatment.

EGCG‐treated tissue samples showed an accumulation of CCN5 protein, which was undetected or minimally detected in untreated samples (Figure 8E‐F), suggesting that tumor growth suppression by EGCG could be mediated through the building up of CCN5 protein in TNBC cells.

Finally, we explored the impact of EGCG‐loaded nanoparticles on the MDA‐MB‐231‐tumor xenograft mouse model. Tumor‐bearing mice were treated with EGCG‐loaded FA‐PEG‐NPs three times a week for 14 days, and tumor volumes were compared with untreated FA‐PEG‐NPs or free‐EGCG‐treated tumors. We considered the treatment endpoint at 14 days because the free‐EGCG effect was first detected after 14 days of treatment (Figure 8B). We found that free‐EGCG and FA‐PEG‐NPs had no impact on tumor growth than the untreated group, while EGCG‐loaded FA‐PEG‐NPs significantly delayed tumor growth (Figure 8G) with no effect on body weight (data are not shown). Collectively, these studies suggest that targeting TNBC by EGCG‐loaded nanoparticles may have substantial therapeutic benefit. However, further studies are warranted.

## 4 DISCUSSION

Over the last decade, several studies, including our laboratory, have revealed the modulatory roles of CCN5 in breast cancer progression.^6^, ^8^, ^9^, ^10^ In addition to suppressing breast cancer cell viability,^7^, ^8^, ^42^ reprograming of MET, a hallmark of reversing cancer stemness mechanism,^43^ is also known to be regulated by CCN5 through TGF‐β‐mediated signaling.^9^, ^13^ Further experimental pieces of evidence have revealed that CCN5 suppresses the expression of the microRNA‐10b, which plays a critical role in microinvasion and metastasis of breast cancer cells ^9^ and induces the expression of the tumor suppressor protein p27.^44^ In a very recent study, the immunomodulatory properties of CCN5 have been reported, according to which CCN5 can enhance the susceptibility of breast tumors to cytotoxic T‐lymphocyte (CTL)‐mediated lysis.^10^ All these factors contribute to the tumor‐suppressive properties of CCN5 against BC. Thus, identifying compounds that can induce the expression of CCN5 in TNBC cells may act as novel therapeutic strategies against BC.

It has been reported that the effectiveness of EGCG against several carcinomas is mediated by promoting cell death and reversing the EMT/cancer stemness.^16^, ^17^ But how EGCG regulates cell death and EMT/stemness in TNBC cells is quite uncertain and warrants further investigation. Hence in the present study, our goal was to investigate the link between CCN5 and EGCG’s pathophysiological roles.

In breast cancers, CCN5 is only detected in non‐invasive cells. In most TNBC cells and pancreatic cancer cells, which are aggressive in nature and mesenchymal type, CCN5 expression is undetected.^7^, ^8^, ^14^, ^31^, ^45^, ^46^ We found that EGCG has the potency to upregulate CCN5 in TNBC and pancreatic cancer cells in a dose‐dependent manner and at the transcription level (Figure 2). These findings suggest the possible involvement of CCN5 in reprograming cell death and MET by EGCG.

The cell viability studies in different breast cancer cell lines showed that EGCG significantly suppresses cell growth in a dose‐dependent fashion with varying IC_50_ values (Figures 3 and 4). We found that the reduced cell viability by EGCG was due to apoptosis via enhancing the CCN5 signal in TNBC cells (Figures 3 and 4). However, how CCN5 specifically promotes apoptosis is unclear, and thus further studies are warranted.

The BTIC/BCSCs, which survive after conventional chemotherapy treatment, typically express unique signature protein markers associated with EMT and stemness^5^, ^47^ and are also known to associate with enhanced clonal growth ability self‐renewal properties.^37^, ^48^ We observed that EGCG, independent of the antiproliferative effect, significantly blocked the self‐renewal or mammosphere‐forming ability of TNBC cells (Figure 5). Simultaneously, EGCG treatment resulted in the drastic inhibition of the expression of mesenchymal and stemness markers. On the other hand, epithelial markers were found to be significantly upregulated (Figure 5). Overall, these findings implicate MET reprograming and self‐renewal in EGCG target in TNBC and potentially in other malignancies.

Although the mechanism of regulation of self‐renewal event by EGCG is unknown, the mechanistic studies found that hrCCN5 protein treatment enhanced the suppressing effect of EGCG on the sphere‐forming ability of TNBC cells, while CCN5 antibody treatment impaired EGCG action (Figure 5). Thus, it is alluring to consider that CCN5 is poised to be a key player in this perhaps currently poorly understood mechanism.

The impact of EGCG in the clinical setup is inconsistent and highly debated, which could be attributed to its very low bioavailability.^49^ Thus, we exploited a nanostructure‐based drug delivery system, known to be an enhancer of bioavailability even at much lower doses than conventional preparations, as the stability of EGCG in the simulated intestinal fluid is significantly improved by encapsulation methods.^40^, ^49^, ^50^ Given the importance of nanoparticle‐based drug delivery, we loaded EGCG into two structurally different nanoparticles established previously ^27^ and determined the efficacy. We found that one of the two EGCG‐loaded nanoparticles (FA‐PEG‐NPs) (Figure 1) was significantly efficient in activating CCN5, reducing cell viability and sphere‐forming ability of TNBC cells (Figures 6 and 7).

Going forward, it was necessary to elucidate the impact of EGCG (free or encapsulated) on TNBC growth in a xenograft model. Our studies found that free‐EGCG treatment significantly delayed the MDA‐MB‐231‐tumor xenograft in nude mice (Figure 8). Since EGCG treatment resulted in proliferating cell nuclear antigen (PCNA) downregulation and activation of CCN5 in tumor tissues, tumor growth inhibition could be mediated via suppressing PCNA, a protein that actively participates in cell cycle regulation and apoptosis,^51^ and CCN5 activation. However, further studies are required to establish this hypothesis.

In concordance with in vitro results of EGCG‐loaded nanoparticles, our in vivo studies demonstrated antitumor efficacy of EGCG‐loaded FA‐PEG‐NPs over free‐EGCG in terms of tumor growth (Figure 8G). Now EGCG‐loaded nanoparticles need to be optimized for safe and on‐target in vivo applications for preclinical and clinical utility.

Based on these novel observations, we may conclude that the reactivation of CCN5 in TNBC by EGCG provides a potentially unique therapeutic strategy for eliminating the residual tumor cells and preventing tumor growth and recurrence.

## CONFLICT OF INTEREST

All authors declare no competing financial interests.

## AUTHOR CONTRIBUTIONS

Conceptualization: S.K.B., A.D. MQ, and SB., Experimental design and Methodology: A.D, IH, P.R., D.D, and A.G., The investigation: P.R., A.D., IH, I.C., A. De., and D.D., Data Analysis: P.R., I.H, A.D., D.D., S.W., S.B., S.K.B, and M.Q., Statistical Analysis: S.G., A.D, I.H., and S.K.B., Original draft: A.D., Writing, Review, and Editing: PR, I.H., S.B., S.K.B., M.Q., and S.W., Supervision: S.K.B., M.Q., and Funding Acquisition: M.Q. SKB, SB.

## DECLARATION OF TRANSPARENCY AND SCIENTIFIC RIGOR

This declaration acknowledges that this paper adheres to the principles for transparent reporting and scientific rigor of preclinical research as stated in the BJP guidelines for Design and Analysis, cell biology techniques, and Animal studies and as recommended by funding agencies, publishers, and other organizations engaged with supporting research.

## Supporting information

Supplementary Material

## Data Availability

The authors affirm that all the supporting data of these studies are available within the articles and Supplementary Information or from the corresponding authors on reasonable request.
