# Supplementary material for: CCN5 activation by free or encapsulated EGCG is required to render triple‐negative breast cancer cell viability and tumor progression
Source: Pharmacol Res Perspect. 2021 Mar 21;9(2):e00753. doi: 10.1002/prp2.753 (PMC7981588; doi:10.1002/prp2.753)
Supplement: Supplementary file 1 — Supplementary Material [file PRP2-9-e00753-s001.pdf]

SUPPLEMENTAL INFORMATION

CCN5 Activation by free or encapsulated-EGCG is required to Render Triple-negative breast cancer cell Viability and Tumor Progression

Amlan Das[1#*], Inamul Haque[1ø], Priyanka Ray[2ø #], Arnab Ghosh[1,3], Debasmita Dutta[4], Mohiuddin Quadir[4], Archana De[1], Sumedha Gunewardena[5], Indranil Chatterjee[1#], Snigdha Banerjee[1,3*], Scott Weir[6] and Sushanta K. Banerjee[1, 3*]

INVENTORY OF SUPPLEMENTAL INFORMATION

1. **Supplemental Data**

   Contain Supplemental Figures S1 and S2 with legends

   - **Figure S1** (Uncropped Western blots)
   - **Table S1**

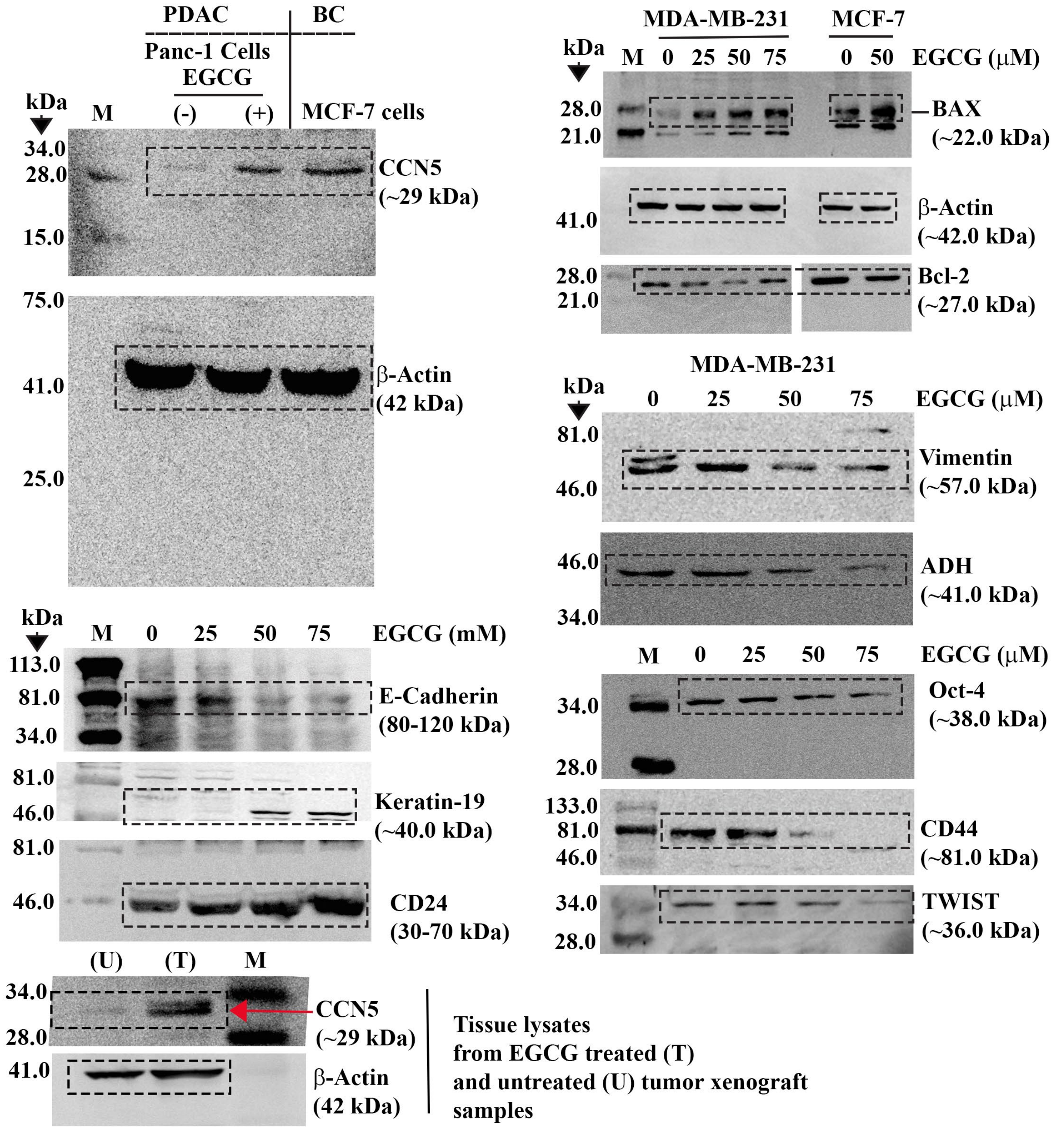

Figure S1 Related to all Figures: Uncropped images of *Western* blot analysis using in these studies.

Table S1: CCN5 and GAPDH (human and mouse) primers for qPCR

Gene Name	Primer Sequences
Human CCN5/WISP-2	**Forward-** 5′-CCTACACACACAGCCTATATC-3′. **Reverse-** 5′-CCTTCTCTTCATCCTACC C-3′
Mouse CCN5/WISP-2	**Forward-** 5′-ATACAGGTGCCAGGAAGGTG-3′ **Reverse-** 5′-GTTGGATACTCGGGTGGCTA-3′
Human GAPDH	**Forward-** 5′-ATGAGAAGTATGACAACAGCC-3′ **Reverse-** 5′-TGAGTCCTTCCACGATACC-3′
Mouse GAPDH	**Forward-** 5′-CTGCTGTCTTGGGTG CAT TGG-3′ **Reverse-** 5-CTCGGCTTGTCACATCT–3′
